# Supplementary material for: Identification of photoperiod-induced specific miRNAs in the adrenal glands of Sunite sheep (Ovis aries)
Source: Front Vet Sci. 2022 Jul 22;9:888207. doi: 10.3389/fvets.2022.888207 (PMC9354845; doi:10.3389/fvets.2022.888207)
Supplement: Supplementary file 1 [file Data_Sheet_1.ZIP › Supplementary file/miRDeep2 file.zip!/miRDeep2 file/Know_Structure/SP42A2/oar-mir-150.pdf]

miRBase precursor : oar-mir-150
Total read count : 734
oar-miR-150 read count : 734
remaining reads : 0

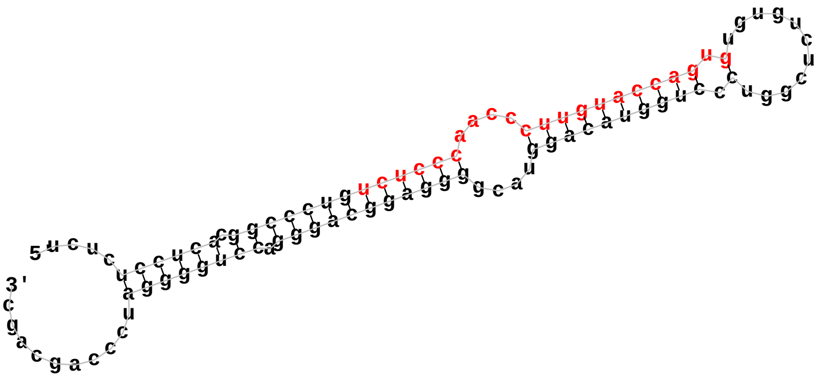

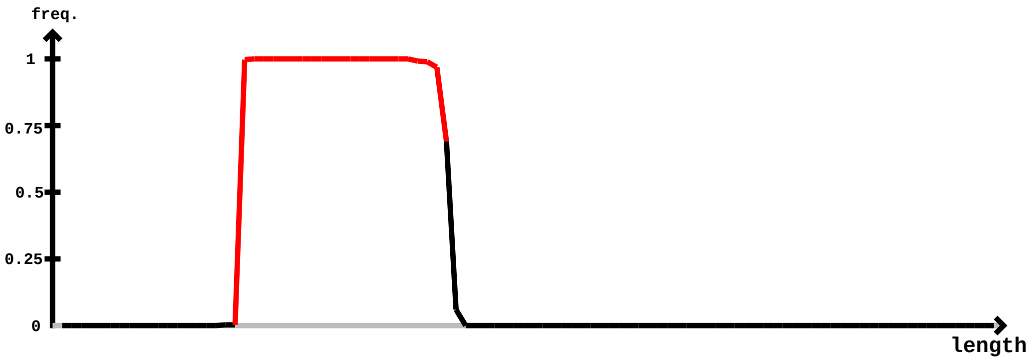


```
                  oar-miR-150
5'- ucucuccucacggcccugucucccaacccuuguaccagugugugucucggucccugguacagguacggggaggcagggaccuggggaucccagcagc   -3'    exp
    ....((((((.(((((((((((((....((((((((((.(...........).))))))))))....))))))))))))).((((((.........    reads     mm        sample
    ................ugucucccaacccuuguacca...........................................................    1         0         seq
    ................ugucucccaacccuuguaccagu.........................................................    1         0         seq
    ..................ucucccaacccuuguacc............................................................    6         0         seq
    ..................ucucccaacccuuguacca...........................................................    1         0         seq
    ..................ucucccaacccuuguaccag..........................................................    15        0         seq
    ..................ucucccaacccuuguaccagu.........................................................    202       0         seq
    ..................ucucccaacccuuguaccagug........................................................    462       0         seq
    ..................ucucccaacccuuguaccagugu.......................................................    44        0         seq
    ...................cucccaacccuuguaccagu.........................................................    1         0         seq
    ...................cucccaacccuuguaccagug........................................................    1         0         seq
```
